# Supplementary material for: Are carbon emissions trading and green financial instruments synergistic? -Comprehensive quantitative research based on content analysis
Source: PLoS One. 2024 Mar 7;19(3):e0298601. doi: 10.1371/journal.pone.0298601 (PMC10919601; doi:10.1371/journal.pone.0298601)
Supplement: S1 Data — (ZIP) [file pone.0298601.s001.zip › Data/地级市Z_F工作报告.zip!/┴╔─■/╙¬┐┌╩╨/└┤╫╘╣1⁄2╓┌║┼í╛┬φ┐╦╩2╛▌═°í┐_2012.docx]

各位代表：

　　现在，我代表市政府向大会报告工作，请予审议，并请市政协委员和列席会议的同志提出意见和建议。

　　一、2011年工作回顾

　　过去的一年，营口在辽宁沿海经济带率先崛起的征程上迈出了坚实的步伐。在省委、省政府和市委的正确领导下，在市人大、市政协的监督支持下，市政府团结带领全市各族人民，围绕“抓升级，促转型，扩增量，惠民生”这一中心任务，拼搏进取，扎实工作，经受住了诸多严峻挑战和考验，经济社会保持了快速健康的发展势头，完成了十四届人大四次会议确定的各项目标任务，实现了“十二五”规划的高起点开局。

　　——经济实力快速提升。预计全年地区生产总值完成1200亿元，增长15%（按可比口径）；地方财政一般预算收入140亿元，增长40%。

　　——发展活力显著增强。固定资产投资完成905亿元，增长30%（按可比口径）；社会消费品零售总额290亿元，增长17.5%；外贸出口34亿美元，增长54%。

　　——社会事业健康发展。社会公共产品均等化水平进一步提高，城乡教育、文化、医疗等公共服务体系日趋完善。

　　——人民生活进一步改善。城镇居民人均可支配收入2.085万元，增长15.5%；农民人均纯收入1.028万元，增长16%。

　　这些发展变化表明，营口率先崛起的态势正在形成，区域竞争中的优势地位进一步巩固，正在发展的快车道上加速前进！

　　（一）优化经济结构，促进三次产业协调发展

　　农业增加值增长6%。粮食生产喜获丰收，总产量达15.3亿斤，增长13.5%，水稻平均单产、最高亩产连续三年双获全省第一。蔬菜、水果、肉蛋奶和水产品产量保持稳定增长。现代农业建设扎实推进。新建设施农业小区379个、1.8万亩；创建省级以上农产品品牌16个，“营口海蜇”成为国家地理标志商标；新增“三品”认定面积11.9万亩，订单农业10万亩。农业机械化综合水平达64.7%。农业产业化取得新突破。龙江福大豆深加工等5个投资10亿元以上农产品加工项目开工建设。连续八年无重大动物疫情发生。举办第三届农业博览会，协议销售额5.2亿元。我市农产品在全国农交会上赢得广泛赞誉。实施东部振兴战略和山区富民工程，扶贫投入5391万元，山区贫困乡镇人均纯收入增加620元。县域经济快速发展。地区生产总值、财政一般预算收入、固定资产投资分别增长28.9%、47.8%、52.8%。

　　规模以上工业增加值增长17%。新增规模以上工业企业152户，新增销售收入超亿元企业80户。中小企业快速发展。民营经济增加值达1050亿元，首次突破千亿大关。产业集群规模不断壮大。镁产品及深加工产业集群实现销售收入630亿元，钢铁及深加工产业集群实现销售收入420亿元，石化、汽保装备产业集群销售收入均超百亿。高新技术产品增加值增长50%，完成企业并购项目10个，投资额超过1亿美元。

　　服务业增加值增长15%。规划建设了六个服务业集聚区，鲅鱼圈海滨温泉旅游、营东新城现代物流、渤海科技城被评为首批“省级现代服务业集聚区”。旅游业发展水平显著提升。双台镇被评为省温泉旅游度假区，虹溪谷温泉旅游度假村等8个项目正式运营。旅游收入145.1亿元，增长27%。组织城市推介、房屋促销活动，房地产业稳健运行。商品房销售额实现230亿元，增长25%。金融环境进一步优化。新增贷款150亿元，金融机构人民币各项贷款余额960.4亿元；新增银行业金融机构4家，达到23家。我市成为国家标准化菜市场改造和现代服务业综合试点市，4个城区全部进入全省经济工作先进城区行列。

　　（二）加大招商引资和项目建设力度，沿海经济带开发开放实现新突破

　　全年新批外商投资企业96家。实际利用外商直接投资11亿美元，增长28%；内联引资实际到位资金400亿元，增长65%。

　　投资10.8亿元的新东北电气精密设备和电力电容、投资10.5亿元的中海油重交沥青、投资10亿元的可口可乐系列饮料等一批项目竣工投产；投资300亿元的五矿（营口）中板大型H钢、投资89亿元的辽宁特钢100万吨不锈钢、投资80亿元的恒力聚酯和香格里拉、维景国际酒店等一批项目开工建设；投资180亿元的中海油LNG、一期投资150亿元的正威高新材料产业基地、投资35亿元的百威啤酒等一批项目正式签约。

　　港口基础设施不断完善。仙人岛航道工程投入使用，成品油及液体化工品泊位部分装卸区竣工投产，集疏运能力显著增强。全年完成货物吞吐量2.6亿吨，增长15.2%；集装箱运量400万标箱，增长20%。营口港连续5年跻身全国十大港口行列。

　　发挥营口港保税物流功能，发展专业化对外贸易，加强镁制品及深加工、汽保装备等出口商品基地建设。外贸进出口总额完成44亿美元，增长50.2%。

　　六个省级重点园区竞相发展。开发区示范作用不断增强，城市功能日臻完善，承载力显著提升，开工亿元以上项目96个，地区生产总值等主要经济指标增幅均在20%以上。北海新区启动区29条、62.8公里的路网工程基本完工，电机产业园开工项目91个，投资总额187.5亿元。沿海产业基地晋升为省级开发区，开工3000万元以上项目200个，二期21.2平方公里基础设施建设全面展开；中小企业创业园区一期南扩工程高效推进，起步区投产企业93户。高新区开工千万元以上项目80个，投产运营企业45户。仙人岛能源化工区能源石化、港口物流、滨海旅游的产业格局已经形成，开工千万元以上项目54个，投产企业10户。沿海新兴产业区入区项目93个，投资总额220.5亿元。沿海经济带多点支撑的发展格局已经形成。

　　（三）加强城乡建设和管理，城乡面貌明显改观

　　全面提升城乡规划管理水平。《营口市城市总体规划》和重点专项规划的编制工作基本完成；制定出台了《营口市城乡规划管理实施细则》，加强规划的协调统一，有力维护了规划的整体性、权威性和严肃性。综合执法、小区物业、道路交通等城市管理向精细化方向推进。列入省规划的7个区镇城镇化试点工作成效显著，农村人居环境不断改善。

　　加快城市建设步伐。河海新西市建设全面提速，西大街一期改造工程竣工；站前北部城区综合改造工程前期工作准备就绪；营东新城初具规模；北海新区、仙人岛新区等新城区基础设施、公共设施建设全面展开。

　　城市功能进一步完善。完成岫水线、庄林线等12条公路建设工程；营口机场前期工作进展顺利，航站区场地回填、机场路等基础工程基本完工；完成大中型水库除险加固和石门水库供水工程，大伙房水库输水配套工程通水试运行，主城区实现24小时稳定供水；实施管网改造和小锅炉房并网等供暖工程，受益面积185万平方米；奥体中心“一场三馆”和文化艺术中心投入使用；垃圾处理厂、营柳河泵站等基础设施工程开工建设。

　　加大生态环境建设力度。实施大规模造林绿化工程，投资43.4亿元，造林面积21.7万亩。农田水利基本建设投入17亿元，农业抗御自然灾害能力进一步增强。实施农村环境连片整治工程，完成了省下达的节能减排任务。城区空气质量优良天数连续4年列全省第一位。大辽河（营口段）水质高于国家重点河流考核标准，摘掉了多年重度污染的帽子。

　　（四）保障和改善民生，社会事业全面进步

　　全年新增实名制就业7.6万人，城镇登记失业率为2.8%，低于全省平均水平。养老保险新增2.9万人，达到47.1万人；医疗保险新增2.6万人，达到104.5万人；工伤保险新增2.9万人，达到29.7万人；生育保险新增1.5万人，达到22.9万人。解决了1.1万名城镇困难居民医疗保险参保问题。企业退休职工养老金、城乡居民低保、在乡复员军人生活补助、农村五保供养、新农合及城镇居民医保补助标准进一步提高。全面实施新型农村养老保险和城镇居民养老保险，参保率达到81%，60周岁以上老人养老金发放率达到100%。

　　市政府承诺的22件重点民生工程全部完成年度计划。积极探索新农合“一卡通”制度，对15家基层医疗卫生机构实施基本药物制度综合改革。完成了8条主干道部分路段和83条街巷路道路道板改造、44个旧住宅小区改造等16项民生工程。地下商业街、残疾人康复中心等5项民生工程进展较快，镜湖公园改造完成前期工作。有力应对盖州小石棚等乡镇近百年来最大的暴雨袭击，保证了人民群众的生命安全，财产损失降到最低，灾后重建工作基本完成。

　　各级各类教育快速发展。营口第二高中、营口实验高中晋升为省级示范性普通高中，大学园二期工程投入使用。教育惠民政策全部落实。发放助学资金1512.9万元，资助贫困学生1.9万名，“控辍保学”工作走在全省前列。规范推进文化体制改革，我市荣获“全国文化体制改革工作先进地区”称号。公益性文化事业蓬勃发展，成功举办了望儿山母亲节、百姓大舞台和首届群众文化艺术节等群众性文化活动，丰富活跃了城乡群众文化生活。列入省规划的乡镇综合文化站建设任务全面完成。开展食品药品安全专项整治，保障了群众饮食用药安全。人口和计划生育服务能力进一步增强，人口出生率5.64％。，人口自然增长率1.42％。。群众体育和竞技体育蓬勃发展，我市在省第六届农民运动会上获得优异成绩。双拥工作再创佳绩，连续五次荣获省双拥模范城称号。开展全国文明城市创建活动，城市文明程度不断提高。

　　国防教育、民兵预备役和人民防空工作不断加强，统计、新闻出版、国家安全、民族宗教、外事侨务、史志、档案、气象、地震等工作进一步强化，慈善、老龄、妇女儿童、残疾人等各项事业取得新进步。

　　（五）加强民主法治建设，促进社会安定和谐

　　坚持依法行政，不断加强法治政府建设。认真贯彻落实市人大及其常委会的各项决议决定，自觉接受人大法律监督、工作监督和政协民主监督，积极听取各民主党派、工商联、无党派人士和各人民团体的意见。全年落实人大代表议案7件。人大代表建议、政协委员提案全部办复。

　　开展“六五”普法，法治城市创建工作取得新进展。城区警务改革顺利进行，社会治安防控体系建设稳步推进；“清网行动”成效显著，打击刑事犯罪和经济犯罪能力显著增强。

　　深入开展“安全生产年”活动，严格排查治理事故隐患，全市安全生产形势总体稳定。妥善办理群众来信来访和投诉，及时解决事关群众切身利益的重点难点问题，维护了和谐安定的社会局面。

　　健全完善惩治和预防腐败体系，积极推进“五大系统”建设，从源头上加大预防腐败的工作力度。工程项目招投标、土地出让、政府采购等公共资源交易步入规范化、制度化轨道，软环境建设水平进一步提高。

　　各位代表：过去的一年，宏观经济环境复杂多变，困难加大，我们积极应对，实现了经济社会又好又快发展，增强了营口人的自信心、自豪感。

　　这些成就的取得，是历届市领导班子工作成效的集中彰显，是市委正确领导的结果，是市人大、市政协和社会各界监督支持的结果，是全市人民共同奋斗的结果。在此，我代表市政府，向辛勤工作在各条战线的同志们，向各级人大代表、政协委员、各民主党派、工商联、无党派人士、各人民团体，向离退休老领导、老同志，向驻营解放军、武警部队官兵和中省直单位，向所有关心、支持营口建设的同志们、朋友们表示衷心的感谢并致以崇高的敬意！

　　回顾一年来的工作，我们清醒地认识到，我市经济社会发展还存在诸多亟待解决的困难和问题。主要表现在：经济总量小，产业层次偏低，产业集中度不高，新兴产业发展滞后；财政收支矛盾突出，对经济社会发展的支撑能力不强；对外开放层次不高，利用外资规模有待进一步扩大；节能减排和环境保护工作压力加大，生产要素供给趋紧；城乡居民收入水平不高，与人民群众的期盼还有差距；维护社会稳定、创新社会管理的任务十分繁重；政府自身建设有待进一步加强。这些问题，我们将采取有力措施认真加以解决。

　　二、2012年工作安排

　　今年是本届政府任期的最后一年。做好今年工作，圆满完成本届政府的各项目标任务，对实现营口在辽宁沿海经济带建设中率先崛起，建设富庶文明幸福新营口至关重要。当前，世界经济复苏乏力，国际贸易增速回落；我国经济增长下行压力和物价上涨压力并存，保持经济持续快速发展的难度加大。面对新情况、新问题，我们既要深刻认识严峻复杂的宏观经济形势，又要充分看到营口仍处在加快发展的重要战略机遇期，我们具备强劲的后发优势、要素资源丰厚的比较优势和内销为主的市场优势；我们的发展框架已经拉开，多点支撑的发展格局已经形成。我们有条件、有能力克服困难，战胜挑战，创造出无愧于时代、无愧于人民的崭新业绩！

　　今年政府工作总体要求是：认真贯彻省委十一届二次全会暨经济工作会议精神，按照市委十一届二次全会的部署，以“坚持科学发展，加速率先崛起”为主题，以加快经济发展方式转变为主线，以招商引资和项目建设为中心任务，深化改革，扩大开放，坚持增量带动结构优化、创新促进产业升级、发展保障民生改善，努力实现经济持续快速健康发展和社会和谐稳定，为建设富庶文明幸福新营口奠定坚实基础。

　　今年全市经济发展的预期目标是：地区生产总值增长15%；固定资产投资增长30%；地方财政一般预算收入增长25%；社会消费品零售总额增长17%；城镇居民人均可支配收入增长14%；农民人均纯收入增长15%。

　　在工作中，要实现一季度“开门红”、上半年“双过半”、全年“两个不低于”的目标。这是省委、省政府对我们的要求，也是我们的奋斗目标，更是我们的庄严承诺！

　　为实现上述目标，重点抓好以下七项工作：

　　（一）全力推进沿海经济带快速发展

　　深入实施以港兴市战略，加大港城联动力度，充分发挥港口对工业化、城市化的牵动辐射作用，以港口功能的完善促进人流、物流和产业的集聚；通过区域经济的快速发展为港口壮大提供强力支撑，构建港为城用、城以港兴的一体化发展格局。

　　做大做强港口实力。加快仙人岛港区疏港铁路、疏港公路、成品油及化工品泊位等基础设施建设。完成保税中心改扩建工程和物流配送中心建设，力争营口港口岸扩大开放获得国家批复，保税港区完成规划设计等前期工作。大力发展金融结算、贸易代理等现代港口服务业，提升港口发展水平。港口货物吞吐量达到2.86亿吨，增长10%；集装箱运量450万标箱，增长13%。

　　对外开放和招商引资是全市经济工作的重中之重。要坚持以园区和产业集聚区为载体，打出主题概念和产业集群的招商旗帜，盯住日韩及香港、台湾、珠三角、长三角等重点地区不放松，不断扩大招商区域，逐步提高国际影响力。全年组织市以上大型招商活动不少于10次。进一步加强与世界500强、行业100强和国内100强企业对接，重点引进一批具有强劲拉动力、核心竞争力的高端装备制造业、高新技术产业、现代服务业项目。

　　今年是我市“重大项目推进年”，要集中精力抓好东旭光电玻璃基板、卓异救生舱、核园核石墨、李锦记无限极生产基地等100个开工、投产项目。全年新开工亿元以上项目260个，10亿元以上项目70个，30亿元以上项目30个；新签约千万元以上项目1000个，包装储备项目1000个，形成项目建设的梯次推进。

　　全市实际利用外商直接投资12亿美元，增长10%；引进域外资金520亿元，增长30%（按可比口径）。加快发展对外贸易，确保外贸出口增长15%。

　　加快重点园区开发建设。开发区要坚持港区协调发展，发挥港区联动优势，加快百万人口新城区建设，主要经济指标增幅确保25%以上。北海新区要加快电机产业园建设，新引进电机项目200个，投产企业20户。沿海产业基地要以高端装备制造产业为核心，重点培育IT和冶金装备、新材料产业集群，引进亿元以上项目100个，投产企业达到100户，积极申办出口加工区；完成中小企业创业园区一期南扩工程，打造国家级中小企业创业孵化基地，投产企业达到150户。高新区要加快渤海科技城二期建设，投产企业达到70户。做大做强仙人岛能源化工区能源石化和港口物流产业，新上一批重大项目。加快沿海新兴产业区高端镁产业化进程，年内形成规模生产。各重点园区实际利用外商直接投资都要达到1亿美元以上。

　　（二）加快转变经济发展方式

　　发展壮大产业集群。抓好镁产品及深加工、石化、电机、汽保装备四个重点产业集群和钢铁及深加工、铝制品及深加工、食品饮品、乐器四个初具规模的产业集群，积极培育输变电、纺织服装、管件阀门等九个初露端倪的产业集群，形成产业集群梯次推进的发展格局。出台扶持政策，对50户创新能力强、市场占有率高、具有较强竞争实力的重点骨干企业，加大引导支持力度，打造一批龙头企业。继续实施中小企业成长工程，着力破解融资难题，促进中小企业快速发展。加快推进公共服务平台建设，完善研发、检测等公共服务体系。举办镁产品及深加工、电机、汽保装备、乐器四个博览会，提升专业园区、产业集群的知名度和影响力。

　　积极培育战略性新兴产业。抓紧完善新兴产业发展规划，加大高新技术产业化基地建设力度，以渤海科技城、鹊鸣湖科技产业园为载体，引进培育50户高成长性的创新型企业，构建以先进制造业为基础、高新技术产业为引领、产业链不断延伸的新兴产业格局。

　　大力发展海洋经济。科学开发利用海洋资源，制定海洋经济发展规划和海洋产业专项规划。以高新技术为引领，加快海洋渔业、船舶工业和港航物流业发展步伐，培育壮大新兴海洋产业，努力构建“蓝色经济”板块。

　　加快发展现代服务业。以规范化、标准化、高端化为方向，提升六个服务业集聚区建设水平。省级服务业集聚区达到5个，服务业增加值增长15%。推进鲅鱼圈、仙人岛现代服务业合作区建设，创建现代服务业体制机制创新示范区。加快大宗商品交易市场和营口港保税物流中心、物流配送中心等项目建设速度，新建、改造国家标准化菜市场15个，完成现代服务业综合试点工作任务。高标准建设鲅鱼圈旅游集聚区、双台温泉度假区等6个温泉小镇，全力打造以白沙湾黄金海岸为核心的滨海景观带，培育开发西大街旅游品牌。旅游收入增长20%以上。加大房地产市场调控力度，促其持续健康发展。以发展现代服务业为重点，进一步壮大城区经济。全市城区生产总值和财政一般预算收入分别增长18%和35%。

　　强化财税和要素保障。狠抓增收节支，加强税收征管，规范非税收入管理，完善财政体制机制；牢固树立过紧日子的思想，压缩“三公经费”，保民生支出，保重大项目支出。合理利用投资基金、企业债券、金融租赁等金融工具，优化信贷结构，保证资金的流动性。引进外埠银行业金融机构3家，新增金融机构贷款170亿元以上。加快企业上市步伐，力争2户企业上市。坚持集约节约用地，提高投资强度。规范土地审批程序，加大建设用地“增减挂钩”、填海造地和闲置土地清理的工作力度，保障建设用地需求。抓好大伙房水库25万立方米水指标调配和17个电力项目建设。

　　加大人才引进力度。加强人才服务平台建设，实施更加有力的吸引、留住人才政策，广泛吸纳汇集国内外著名高校、科研院所、知名企业的专家学者、管理人员等高层次、高技能人才。全年引进海外研发团队7个，引进高层次人才500名。采取产业移民、安居移民等措施，努力扩大人口规模。

　　（三）全面提升农业和县域经济发展水平

　　大力发展现代农业。新建设施小区130个，新增订单农业10万亩，扩建蔬菜基地1.4万亩，建设蔬菜基地质量安全检验检测站11个。农业机械化综合水平提高3.8个百分点。

　　粮食生产面积稳定在146万亩以上，提升水果、水产、绒山羊和柞蚕等特色产业规模和效益。抓好大石桥优质稻米、盖州苹果“一县一业”建设，开展十佳“专业乡、专业村、龙头企业和农民专业合作社”创建活动。新增“三品”认证30个，争创省以上品牌20个。

　　加快农业产业化步伐。新上千万元以上农产品加工项目20个，继续引进投资10亿元以上的农产品加工项目，推动首兴木糖醇、津大生态肥料等7个重点项目建设投产。

　　大力发展县域经济。盖州市、大石桥市要按照中等城市规模高标准抓好县城规划，加快服务业集聚区规划建设，下力量抓好县城开发和城市化样板乡镇、试点村建设。县域生产总值、固定资产投资、地方财政一般预算收入分别增长27%、40%、35%。

　　全力推进东部振兴。加大投入力度，加快东部贫困山区基础设施建设、生态环境保护、产业和社会事业发展。解决11.2万人饮水安全问题。气象灾害防御体系实现东部山区全覆盖。推广适宜山区作业的农业机械，为12个重点贫困乡镇的36个贫困村各建一个农机专业合作社。加大扶贫开发力度，161个贫困村农民人均纯收入增加1000元。

　　加强农业基础设施建设。抓好6个乡镇的节水灌溉项目，完成11万亩优质水稻水渠节水改造；治理中小河流30公里、病险水库3座、水土流失面积10万亩。加强农村公路建设，实施一事一议财政奖补政策，新建农村公路212公里。

　　开展宅基地、林权抵押贷款试点工作，推进“十星级文明户”道德信贷金卡工程，努力破解农村发展资金难题。抓好农业保险和林业保险，为农民增收提供保障。

　　（四）加强城市和生态环境建设

　　进一步加强城市规划管理。完成《营口市城市总体规划》报批工作；抓好城乡建设与土地利用、基础设施与产业发展规划的衔接，实现主城区控规全覆盖；编制城市天际线、色彩、交通等专项规划，提升单体建筑和景观设计水平。明确城镇规划定位，做好广场、商业街区及绿化、亮化设计。严格规划审批实施，维护规划的严肃性和权威性。

　　坚持高水平改造老城区，高标准建设新城区。加快沿海城市带、产业带、生态带建设，推进老城区与营东新城、大石桥；盖州老城区与北海新区；开发区与仙人岛新区一体化发展，构建“一带三轴”的大城市发展格局。加大老城区改造力度。年内老城区120个老旧小区全部改造完毕；对15条道路实施综合改造，对部分街路、广场、公园实施绿化美化亮化；对市区部分交叉路口实施渠化改造，缓解交通压力。加快新城区建设，年内营东新城基本建成，北海新区初具形象。

　　实施城市精细化管理。针对城市管理存在的突出问题，进一步理顺管理体制，下移管理重心，提高管理效能。建立综合执法、环卫、园林、市政等多部门联动的管理机制，实现管理全覆盖。加强小区物业管理，重点做好改造后小区和保障性住房小区的管理工作。加大市容市貌综合整治力度，严格规范广告牌匾和建筑工地秩序，严格道路交通管理，营造整洁有序的市容环境。启动数字营口建设，不断完善城市信息化基础设施，建立城市管理的长效机制。

　　加强基础设施建设。加快机场建设进度；完成大伙房水库输水配套工程、高铁快速通道和三个高铁站前广场建设；继续推进燃气气源厂改扩建和管网改造工程；实施东部污水处理厂中水回用工程，加快生活垃圾处理厂建设；新建4条机动车检测线。

　　改善城乡环境。争创全国森林城市，开展大规模造林绿化，实施青山工程。造林29.2万亩，植树850万株，封山1.9万亩，完成蟠龙山、白狼山等8处矿山治理示范工程。加大大石桥市环境污染治理力度，确保年内初见成效。实施51个村环境连片整治工程，建设无害化卫生厕所1万座。抓好十项节能工程，实施重点节能资源综合利用项目30项，建设省内一流的再生资源分解加工中心，完成省下达的节能减排任务，争创全国卫生城市。

　　（五）倾力保障和改善民生

　　完成上年结转的重点民生工程，新启动实施15件重点民生工程。

　　努力增加城乡居民收入。完善工资集体协商制度，引导企业提高职工工资水平；加大棚户区改造力度，提高居民财产性收入；加强对低收入困难群体的补贴和救助，进一步提高最低工资标准、企业退休人员基本养老金标准、城乡低保标准、农村五保供养标准和重点优抚对象生活补助标准；完善困难群众基本生活价格补贴机制；落实涉农补贴政策；加强市场监管，努力保持居民消费价格基本稳定。采取措施预防和解决农民工工资拖欠问题，确保农民工工资按时足额发放。

　　稳定和扩大就业。全年新增实名制就业7.8万人，城镇登记失业率控制在3%以内。做好高校毕业生的就业工作，对困难家庭大学生就业实行兜底安置；加强就业困难群体的就业援助工作，确保零就业家庭动态为零；强化普惠制就业培训，实名培训城镇下岗失业人员1.7万人，培训农村剩余劳动力1.7万人。

　　完善社会保障体系。扩大新农保和城镇居民养老保险覆盖面，参保率达到90%以上，60周岁以上老人养老金确保按时足额发放。做好医疗、工伤等保险扩面和保费征缴工作。提高城镇居民基本医疗保险和新农合补助标准，完善医疗保险市级统筹和门诊统筹。加强应急救助和城乡医疗救助，健全优抚保障机制和社会救助体系。加快养老服务体系建设，促进老年人生活水平不断提高。加强住房保障体系建设，进一步改善低收入家庭的住房条件。

　　（六）大力发展社会事业

　　坚持教育优先发展。积极发展公办幼儿园，规范发展民办幼儿园，新建、改扩建幼儿园20所。推进义务教育学校标准化建设，实施义务教育均衡发展示范县（区）和基础教育强县（区）工程，促进城乡教育均衡发展。大力发展职业教育，提高技能型人才培养质量。努力发展高等教育，积极推进本科大学申办工作，加快规划大学园北海新校区。

　　发展文化事业和具有营口特色的文化产业。实施5项文化惠民工程；新建两个市民活动中心；完成县城数字影院建设；继续办好望儿山母亲节和国际海滨温泉旅游节，丰富城乡群众文化生活。深度挖掘文化资源，加快发展文化产业。高起点推进金牛山国家考古遗址公园、盖州古城、熊岳古城和玉石文化博览园、乐器产业园、创意产业孵化园建设，打造营口特色文化产业品牌。

　　促进卫生、体育等各项事业全面发展。加强基层医疗卫生体系建设，扩大基本药物制度实施范围。做好公立医院改革试点工作，着力提高医疗技术水平。强化食品、药品监管，确保饮食用药安全。实施农民体育健身工程，为20个乡镇和100个村各安装一套健身器材。认真做好“十二运”筹备工作。加强人口和计划生育工作，开展“富庶文明幸福营口家庭”创建活动。加强诚信营口建设，扎实推进全国文明城市创建工作，以创促建、以创促改，不断提升城市文明程度。

　　强化国防教育和民兵预备役、人民防空工作，积极开展双拥共建活动。进一步做好统计、新闻出版、国家安全、民族宗教、外事侨务、史志、档案、气象、地震等各项工作。积极发展慈善、老龄、妇女儿童、残疾人、未成年人等各项事业。

　　加强和创新社会管理。充分发挥工会、共青团、妇联组织等社会各方面的协同作用和群众参与社会管理服务的主体作用，形成规范化、制度化的社会管理体系。加强基层基础建设，进一步强化城市、农村社区服务功能，完善服务网络。

　　深入开展“六五”普法活动，认真做好经常性矛盾纠纷排查调处工作。强化社会治安综合治理，推进公安信息化和平安和谐社区建设。积极推进户籍制度改革，优化流动人口服务管理，加强特殊人群管理。完善预防、管理和打击相结合的治安防控体系，争创全国综治工作优秀市。坚持用群众工作统揽信访工作，充分发挥信访接待大厅的作用，建立联合接访机制，提高信访工作效率。坚持领导干部大接访制度，坚决纠正损害群众利益行为。

　　加强安全生产标准化建设，强化安全生产责任制，坚决遏制重特大事故发生。加强校车安全管理。完善防灾减灾预警系统。健全突发性公共事件应急管理机制，强化政府应急管理职能，提高应急处置能力。

　　（七）努力建设人民满意政府

　　面对复杂形势和繁重任务，我们必须牢固树立敢于担当、敢于负责的精神，提高政府部门的执行力、公信力。

　　提振发展激情。各级政府和广大公务员要在“坚持以人为本，加速率先崛起”主题实践活动中，当表率、做先锋。必须目标高远，切实增强赶超先进的信心和勇气；必须抢抓机遇、乘势而上，采取超常举措，务求干则必成；必须强化时间观念、效率意识，力求最短时间取得最佳效果；必须加强学习，提高本领，使政府的工作水平和行政管理能力与营口经济社会发展相适应。

　　坚持把依法行政贯穿于政府工作的全过程。要自觉接受人大法律监督、工作监督和政协民主监督，全面加强行政执法规范化建设，建立规范、顺畅、高效的行政秩序。进一步转变政府职能，认真解决好行政管理越位、错位、不到位问题。

　　实行严格的目标责任管理。强化监督考核、行政问责，严格规范公共资源管理，严肃查处违法违纪案件，坚决纠正不正之风，建设一支风清气正、廉洁高效的公务员队伍。

　　各位代表：历史激励着我们，时代召唤着我们，营口243万人民期待着我们。让我们在市委的坚强领导下，在市人大、市政协的监督支持下，不停顿，不懈怠，不放松，恪尽职守，不辱使命，以更加优异的成绩迎接党的十八大胜利召开！
